# Conventional Versus Accelerated Collagen Cross-Linking for Keratoconus: A Comparison of Visual, Refractive, Topographic and Biomechanical Outcomes

**DOI:** 10.2174/1874364101711010262

**Published:** 2017-08-29

**Authors:** Jyh Haur Woo, Jayant Venkatramani Iyer, Li Lim, M Htoon Hla, Jodhbir S Mehta, Cordelia ML Chan, Donald TH Tan

**Affiliations:** 1 General Cataract and Comprehensive Ophthalmology, Singapore National Eye Centre, Singapore, Singapore; 2 Corneal and External Eye Disease Service, Singapore National Eye Centre, Singapore, Singapore; 3 Singapore Eye Research Institute, Singapore, Ophthalmology and Visual Sciences Academic Clinical Program, Duke-NUS, Singapore, Singapore

**Keywords:** Cross linking, Keratoconus, Corneal biomechanics, Collagen, Topographic, Biomechanical outcomes

## Abstract

**Objective:**

The aim was to compare the visual, refractive, topographic and biomechanical outcomes in patients with progressive keratoconus treated with either conventional or accelerated crosslinking at one year follow up.

**Methods:**

It is a prospective, non-randomised interventional study of 76 patients who underwent conventional (CXL; 3mW/cm^2^ for 30 minutes) or accelerated cross linking (KXL; 30mW/cm^2^ for 4 minutes) for progressive keratoconus. Baseline and postoperative visual acuity, manifest refraction, corneal topography, pachymetry, endothelial cell density and biomechanical parameters of corneal hysteresis and corneal resistance factor were evaluated and compared.

**Results:**

The 2 groups were comparable in terms of uncorrected and best corrected visual acuity and spherical equivalent. Both groups showed no significant increase in K1, K2 and Kmean from baseline at 12 months. There was also no difference between the CXL and KXL group for postoperative corneal topography as well as central and minimal pachymetry up to 12 months. There was a significant increase in both corneal hysteresis (0.62mm Hg, P=0.04) and corneal resistance factor (0.91mm Hg, P=0.003) in the KXL group at 12 months but not in the CXL group. There was no significant endothelial cell loss throughout follow up in both the groups.

**Conclusion:**

We have established comparability of the 2 protocols in stabilizing the progression of keratoconus. Our findings also suggested an added biomechanical advantage of accelerated crosslinking at 1 year follow up.

## 1 INTRODUCTION

Collagen crosslinking is an established treatment for keratoconus and other ectatic corneal disorders, with proven efficacy in slowing or halting disease progression [1-3]. In the procedure, the induction of cross-links between intrastromal collagen fibrils by photosensitizer riboflavin and ultraviolet A (UV-A) irradiation confers added corneal rigidity and strength, thereby stabilizing the ectatic process. Since the first report of crosslinking in the treatment of progressive keratoconus in 2003 [4], there has been extensive research on expanded indications and various modifications of the procedure evolved from the original “Dresden protocol” for conventional crosslinking, including accelerated protocols with increased irradiance over shorter duration, treatment through an intact epithelium (“epi-on”), as well as combination therapy with intracorneal ring segments and refractive surgery across different crosslinking platforms [2, 5].

Of interest, accelerated or high-fluence protocols present a promising alternative to the time-consuming conventional crosslinking. The potential advantages include reduced exposure time, better patient compliance and lower infection risk. According to Bunsen-Roscoe’s law of reciprocity, an increased intensity of UV-A irradiation coupled with reduced exposure time theoretically delivers a total energy dose to the tissue equivalent to that in conventional treatment, with similar biological effect [6, 7]. *Ex-vivo* experiments on porcine corneas have yielded similar outcomes on biomechanical properties with high energy and short irradiation time settings compared to standard protocol [6]. However, others have reported reduced treatment efficiency, postulated to be a result of intrastromal oxygen diffusion capacity and increased oxygen consumption associated with higher irradiances [7], and limited biomechanical strengthening beyond irradiance of 50 mW/cm^2^ and exposure time of less than 2 minutes [8] in animal tissue. There has been no significant difference in corneal stiffness between human eyes crosslinked with high and low intensity protocols *ex-vivo* [9]. Several clinical studies have suggested that the effectiveness of accelerated crosslinking is comparable to conventional treatment with similar safety profiles [10-19]. However, the lack of a uniform protocol and differing research methodologies have made comparisons between these studies difficult and more evidence is needed to confirm the efficacy of accelerated crosslinking in spite of its purported advantages over standard protocol.

This paper aims to compare the visual, refractive, topographic and biomechanical outcomes in patients with progressive keratoconus who were treated with either conventional or accelerated crosslinking.

## 2 MATERIALS AND METHODS

Two prospective interventional studies of patients who underwent conventional and accelerated cross linking for progressive keratoconus were conducted consecutively in Singapore National Eye Centre, Singapore from 2008 to 2015. The studies were approved by the SingHealth Centralised Institutional Review Board (CIRB) and adhered to the tenets of the Declaration of Helsinki. Informed consent was obtained from all the participants.

The studies were registered with ClinicalTrials.gov: NCT 01123057, NCT02638376.

An initial detailed screening of all potential study subjects was first performed to determine suitability for crosslinking. Seventy six patients with 76 eyes, who were at least 18 years of age with documented progressive keratoconus based on topographic (increase of 1.0D or more in the steepest keratometry), pachymetric (reduction in minimal corneal thickness of 5% or more), visual acuity and refractive changes (increase in cylinder of more than 1.00D or spherical equivalent of more than 0.50D) over at least 6 months, were included in the studies. Eyes with corneal pachymetry of less than 400µm at the thinnest point, endothelial cell density of less than 2000 cells/cm^2^, corneal scarring, nystagmus or any motility disorders which prevented a steady gaze during examination and imaging, and other significant ocular disease were excluded. Patients who have a history of autoimmune disorders or were pregnant or breastfeeding at the time of recruitment were also excluded. All patients would have discontinued their rigid gas permeable (RGP) contact lens wear for at least 3 days before the screening visit.

All subjects underwent a complete ophthalmic examination which included uncorrected (UCVA) and best corrected visual acuity (BCVA) based on the logMAR chart, manifest refraction, slit lamp and dilated fundoscopy. Corneal topography was performed using the Pentacam conventional Scheimpflug system (Oculus Optikgerate GmbH, Munchholzhauser str.29, 35582 Wetlar, Germany). The values representing the flat, steep and mean keratometry (K1, K2 and Kmean) were recorded. The central and minimal pachymetric measurements were also derived from the Pentacam system. The endothelial cell density (ECD) was assessed using a non-contact specular microscope, Konan Noncon Robo SP8000 (Konan Medical, Hyogo, Japan). The biomechanical parameters of corneal hysteresis and corneal resistance factor were measured using a dynamic bidirectional applanation device (Ocular Response Analyzer, Reichert Ophthalmic Instruments, Depew, NY, USA). These investigations were performed at baseline and on follow up.

All crosslinking procedures were performed by three surgeons (LL, JM, CC) under topical anaesthesia and sterile conditions in the operating theatre. In conventional crosslinking (CXL), corneal pachymetry was first performed before the procedure. The corneal epithelium was then partially removed from a 9.0 mm treatment zone using a smooth spatula. One drop of isotonic riboflavin 0.1% with dextran 20% solution (MedioCROSS D, Medio-Haus-Medizinprodukte GmbH, Kiel, Germany) were instilled every 2 minutes for 30 minutes (15 drops). Thereafter, the corneal thickness measurement was repeated and if less than 400µm, 2 drops of hypotonic riboflavin 0.1% solution (MedioCROSS H, Medio-Haus-Medizinprodukte GmbH, Kiel, Germany) was instilled every 10 to 15 seconds until the corneal thickness was at least 400 µm. The patient’s eye was then positioned under the UV illumination device (UV-X, Peschke Meditrade GmbH, Huenenberg, Switzerland), taking care to ensure that the beam diameter was within the treatment zone avoiding the limbal area and a 5cm distance between beam aperture and eye. The riboflavin solution was then instilled every 2 minutes during the illumination process at an irradiance of 3mW/cm^2^ for 30 minutes (total energy: 5.4J/cm^2^). In accelerated crosslinking (KXL), 1 drop of dextran-free riboflavin 0.1% solution (VibeX Rapid, Avedro, Inc., Waltham, Massachusetts, MA, USA) was instilled every 2 minutes for 10 minutes after epithelial removal. The eye was then rinsed thoroughly with balanced salt solution and aligned under the UV illumination system (KXL, Avedro, Inc., Waltham, Massachusetts, MA, USA), following which irradiation was conducted at 30 mW/cm^2^ continuously for 4 minutes (total energy: 7.2J/cm^2^). Key differences between the 2 protocols have been summarised in Table **1**. For both protocols, a bandage contact lens was applied post-procedure and the patients were started on topical antibiotics (moxifloxacin hydrochloride 0.5%) and steroids (prednisolone acetate 0.12%), which were tapered at 1 month after complete epithelial healing. All patients were reviewed at 1 day, 1 week, 1 month, 3 months, 6 months and 12 months after the procedure.

All statistical analyses were conducted using the Statistical Package for the Social Sciences V.17.0 (SPSS Inc, Chicago, Illinois, USA). Two samples independent T-test and paired T-tests were performed for normally distributed variables, and nonparametric tests were used if variables are not normally distributed. A probability of less than 5% (p<0.05) was considered statistically significant.

## 3 RESULTS

### 3.1 Baseline Characteristics (Table **2**)

We included 76 eyes from 76 patients in the studies, of which 29 eyes underwent conventional crosslinking while 47 eyes underwent accelerated crosslinking. The mean age (+/- SD) of the patients was 29.16 +/- 7.3 years and 27.88 +/- 7.1 years in the CXL and KXL group respectively. The majority of patients in each group were male, with 37 patients (78.7%) in the KXL group and 21 patients (72.4%) in the CXL group. The patients were also predominantly Chinese (CXL, N=17, 58.6%; KXL, N=31, 66.0%). The mean follow up period was 13.1 months in the CXL group and 12.2 months in the KXL group. There was no statistical difference between the 2 groups in terms of demographics, preoperative visual acuity, refraction, topography, pachymetry and biomechanical parameters. However, the KXL group had significantly higher preoperative endothelial cell density than the CXL group.

### 3.2 Visual Acuity and Refractive Outcomes

In the CXL group, there was significant improvement in the UCVA from baseline of 0.13 (P= 0.003) and 0.11 (P=0.017) at 3 months and 6 months respectively. For BCVA, the subjects in the CXL group showed significant improvement from baseline of 0.11 (P=0.037) at 12 months. In the KXL group, there was no statistically significant change in UCVA throughout follow-up, with improvement in BCVA seen at 6 (0.06, P=0.006) and 12 months (0.08, P=0.004). There was no statistically significant difference in the change in both uncorrected and best corrected LogMAR visual acuity from baseline between the 2 groups throughout follow up, except for UCVA at 3 months (CXL: -0.13; KXL: -0.01; P = 0.01).

In terms of refractive outcomes, within the CXL group, there was no change in spherical equivalent (SE) throughout follow up but in the KXL group, the subjects had a significantly more myopic SE compared to baseline at 1 month (-0.72D, P=0.046) and 3 months (-0.65D, P=0.019) only (Table **3**). There was no significant change in SE from baseline at 12 months in the KXL group. There was no significant difference in the change in spherical equivalent between the 2 groups up to 12 months, except for the measurement at 1 month (CXL: 1.01D; KXL:-0.72D; P = 0.008).

In the KXL group, there was a statistically significant deterioration in cylinder error from baseline at 1 month (-0.46D, P=0.013), 3 months (-0.46D, P=0.009), 6 months (-0.41D, P=0.026) and 12 months (-0.55D, P=0.002). (Table **3**) Subjects in the CXL group had an improvement in cylinder correction at 1 month (1.01D, P=0.048) only. The KXL group also had a significant worsening in cylinder error at 1 month (CXL: 1.01D; KXL: -0.46D; P = 0.008) and 3 months (CXL: 0.80D; KXL: -0.46D; P = 0.025) compared to the CXL group, but this trend was not observed at subsequent follow ups at 6 and 12 months.

### 3.3 Topographic Outcomes

**Fig. (1):**
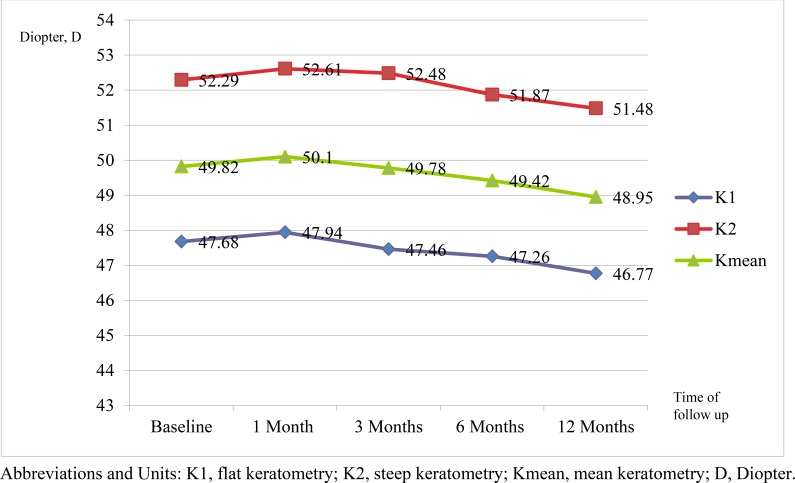
Figure showing K1, K2 and Kmean with time in the CXL group.

**Fig. (2):**
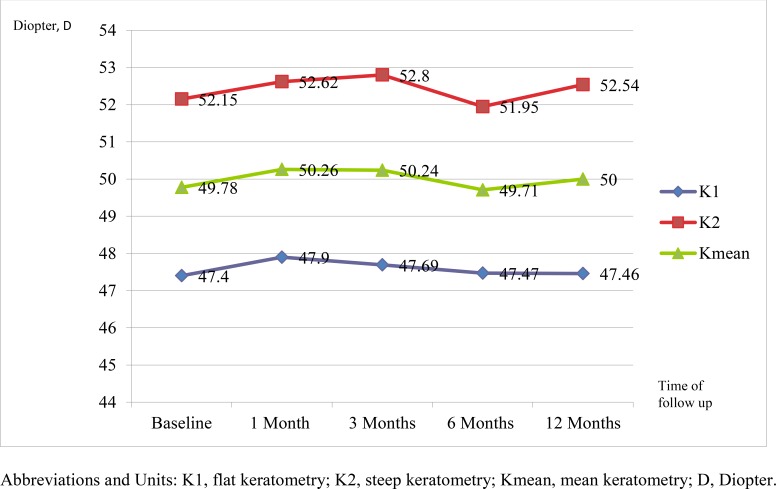
Figure showing K1, K2 and Kmean with time in the KXL group.

There was no significant increase for K1, K2 and K mean compared to preoperative measurements at 12 months for both CXL (Fig. **1**) and KXL (Fig. **2**). Similarly, there was no difference between the CXL and KXL group for the change in postoperative K1 (CXL:-0.39D; KXL: -0.13D; P=0.352), K2 (CXL: -0.13D; KXL:-0.21D; P=0.829) and Kmean (CXL:-0.13D; KXL: -0.17D; P=0.626) values at 12 months.

### 3.4 Pachymetric Outcomes

Within the CXL group, there was a significant reduction in central corneal thickness from baseline measurements at 1 month (-8.10 µm, P=0.016), and a significant reduction in minimal corneal thickness was seen at 1 month (-8.97 µm, P=0.041) and 3 months (-9.34 µm, P=0.043). There was no significant change in both measurements from baseline at 12 months. For the KXL group, both the central and minimal corneal thickness measurements were reduced at 1 month (central, -8.03 µm, P=0.044; minimal, -12.08 µm, P=0.005), reaching a maximum at 3 months (central, -19.56 µm, P<0.001; minimal, -21.00 µm, P<0.001) before recovering at 6 months (central, -8.56 µm, P=0.015; minimal -7.97 µm, P=0.026). However, at the 12-month follow up, there was no statistically significant difference in both measurements from baseline.

There was no significant difference in the change in central and minimal corneal thickness with time between the 2 groups, with the exception being the central corneal thickness measurement at 3 months, in which the KXL group showed a greater reduction than the CXL group (CXL: -7.28 µm; KXL -19.56 µm; P=0.042).

### 3.5 Biomechanical Outcomes (Figs **3** and **4**)

We found a significant improvement in corneal hysteresis from baseline (0.62 mm Hg, P=0.04) in the KXL group at 12 months. In the KXL group, the corneal hysteresis increased from 8.23 mmHg to 8.81 mmHg at 12 months (P=0.04). In the CXL group, the corneal hysteresis changed from 7.82 mmHg at baseline to 7.7 at 12 months. However, this difference of 0.12 mmHg was not statistically significant (P = 0.621).

There was a statistically significant increase from baseline measurement of corneal resistance factor for those within the KXL group at 1 month (0.61 mm Hg, P=0.034), 6 months (0.78 mm Hg, P=0.013) and 12 months (0.91 mm Hg, P=0.003). For the CXL group, there was no significant change in the corneal resistance factor between baseline and up to 12 months.

### 3.6 Endothelial Cell Density

There was no statistically significant reduction in endothelial cell density for both the CXL and KXL groups at all time points throughout follow up. In the CXL group, the baseline ECD was 2860 cells/mm^2^ while that at 12 months was 3002 cells/mm^2^ (P=0.05). Similarly, in the KXL group, the preoperative ECD was 3146 cells/mm^2^ while that at 12 months was 2912 cells/mm^2^ (P=0.06)

### 3.7 Complications

Two patients in the CXL group developed late onset deep stromal scarring and this has been published elsewhere [20]. Notably, the stromal scar formation occurred away from the visual axis and did not affect the final best corrected visual acuity in both cases. There were no long term complications in the KXL group.

## 4 DISCUSSION

To date, there is no consensus on the safety and efficacy of accelerated high fluence collagen crosslinking compared to conventional protocol. While some studies have reported similar results in terms of visual acuity, refractive and topographic outcomes, a few have found the effect of accelerated crosslinking on disease stabilization to be limited. (Table **4**) Notably, current evidence is based mostly on studies with small sample sizes, which may be underpowered to detect significant differences. Differing irradiance used for accelerated crosslinking protocols (ranging from 7 to 30 mW/cm^2^), ultraviolet radiation systems, postoperative regimens and follow-up duration further confound any meaningful comparison between studies.

In the present study, the CXL and KXL groups showed improvement in BCVA of 0.11 and 0.08 LogMAR units respectively at 12 months compared to baseline. Shetty *et al*. in a prospective randomized interventional study of 138 eyes with keratoconus which underwent crosslinking at radiance of 3, 9, 18 or 30mW/cm^2^, found that while there was an improvement in the corrected distance visual acuity in all groups at 12 months, the change was not significant in the 30mW/cm^2^ group and the most improvement occurred in the 18mW/cm^2^ group [12]. However, no such intergroup difference was found in our study. Various authors have reported a reduction in spherical equivalent and cylinder error in both accelerated and conventional crosslinking, but with no significant difference between the 2 groups [10, 17, 18]. Our study showed no difference between the 2 groups at 12 months when the change in spherical equivalent value was considered. There was a significant deterioration in mean cylindrical error in the KXL group at all time points throughout the follow up period. However, for the CXL group, the improvement in cylindrical error was only significant at 1 month. (Table **3**) There was no difference between the 2 groups beyond 3 months.

We did not find any differences for K1, K2 and Kmean values after 1 year of follow up. Our results are similar to existing data in which there is generally no significant difference between accelerated and conventional protocols in terms of topographic change [11, 13, 15, 18]. Not all authors agree on that standard and high fluence protocols change corneal topography to the same extent. Shetty *et al*. noted that the flattening effect of crosslinking was reduced with higher irradiation and shorter treatment duration [12]. A retrospective analysis of 131 eyes with progressive keratoconus by Brittingham *et al*. [16] even showed a negative effect on topographic outcome at 1 year, with the mean change of -0.76D with standard protocol versus +0.72D in the accelerated group.

Two studies have observed that both central and minimal corneal thickness measurements were reduced to a lesser extent in accelerated high-fluence crosslinking compared to conventional protocol [12, 18]. However, we did not find any difference between the CXL and KXL group in terms of change in central or minimal corneal thickness, which were reduced at 1 month before recovering to near preoperative levels at 12 months. Interestingly, the values for both central and minimal corneal thickness were lowest at 3 months for the KXL group while the cornea was thinnest between 1 and 3 months for the CXL group. Jordan *et al*. in prospective *in *vivo** confocal microscopy study of corneal microstructural changes after crosslinking for keratoconus, showed the complete absence of stromal keratocyte nuclei in 86% of corneas at 1 month, while anterior stromal edema with hyper-reflective cytoplasm and extracellular lacunae in a honeycomb-like appearance may persist at 3 months postoperatively [21, 22]. These anterior stromal changes were also more pronounced in accelerated crosslinking compared to conventional crosslinking [23]. The reduction in corneal thickness between 1 and 3 months may be a result of progressive re-epithelization and compaction of stromal lamellae after crosslinking. There is also evidence that pachymetry with Scheimpflug imaging system may underestimate corneal thickness in the early postoperative period due to stromal haze and changes in reflectivity [24].

**Fig. (3):**
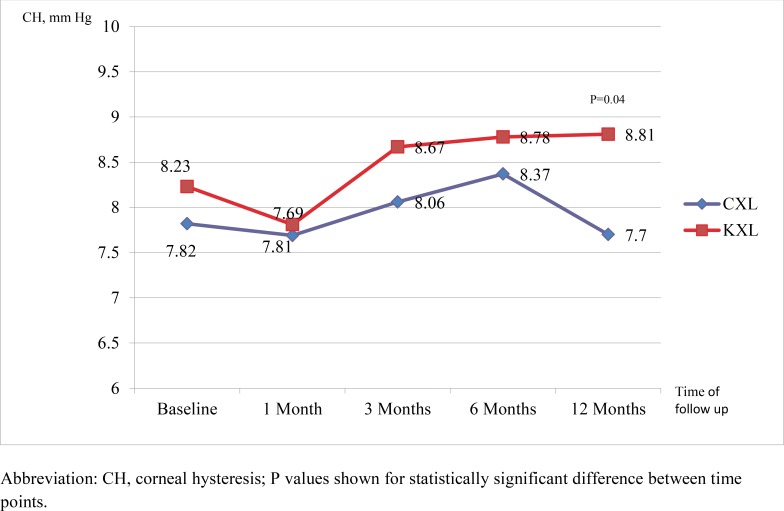
Comparison of corneal hysteresis with time between the CXL and KXL group.

**Fig. (4):**
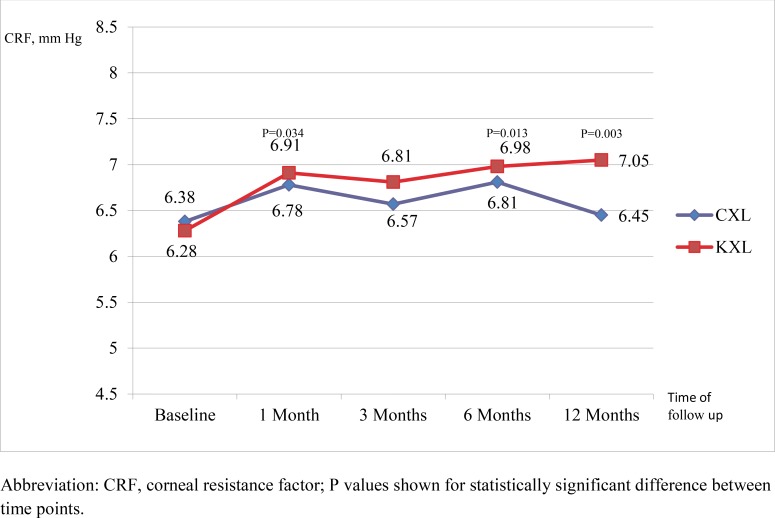
Comparison of corneal resistance factor with time between the CXL and KXL group.

We have demonstrated a positive change in both corneal hysteresis and corneal resistance factor, as measured by the ocular response analyzer, associated with accelerated crosslinking up to 12 months after the procedure (Figs. **3** and **4**). This is in contrast to the findings of previous studies on biomechanical properties of the cornea after crosslinking, which reported no change in corneal hysteresis and corneal resistance factor [25-28] To the best of our knowledge, this has not been reported before. Various *in vitro* animal studies [27, 29] and *ex vivo* trials on human eyes [30, 33] have provided strong evidence of increased corneal rigidity and resistance to enzymatic digestion with crosslinking [34]. Comparative trials have not shown any difference in biomechanical parameters between conventional and high-fluence, short duration protocols [13, 15, 18]. An *ex vivo* human corneal study by Kanellopoulos *et al*. also found the biomechanical effect of CXL studied by resistance to enzymatic digestion in human corneas to be comparable between irradiances of 9, 18 and 30 mW/cm^2^ [35]. However, before the current study, these effects have never been replicated in clinical studies, likely due to the differences in quantification of corneal rigidity and the high variability in resistance to deformation in irregular keratoconic corneas [27]. We postulate that the improved biomechanical effects demonstrated in accelerated crosslinking may be attributed to possible differences in UV radiation beam profile between the 2 protocols, though this has to be further validated.

This paper may be limited by a lack of sufficient statistical power due to small patient cohorts. There was also no randomization as the 2 groups of patients were treated consecutively. For practical purposes, some of our patients could only stop the use of their RGP contact lens 3 days from the day of evaluation and this may limit the reliability of the keratometric results. Our analysis was limited to the 1 year outcomes even though the CXL group had a longer post-treatment duration. Lastly, we did not have data with regards to the demarcation line which may further substantiate the comparison of treatment efficacy between the 2 protocols.

## CONCLUSION

In conclusion, our study has strengthened the evidence on the efficacy and safety of accelerated high-fluence crosslinking compared to conventional crosslinking. Both protocols were effective in stabilizing the keratoconus at 1 year follow up. Our findings also suggested an added biomechanical advantage of accelerated crosslinking at 12 months. Larger prospective randomized controlled trials with longer follow up are necessary to confirm the long term safety and efficacy of accelerated crosslinking.

## Figures and Tables

**Table 1:** Conventional and accelerated crosslinking protocols.

**Conventional Crosslinking**	**Protocol**	**Accelerated Crosslinking**
Yes	**Removal of epithelium**	Yes
Isotonic riboflavin 0.1% with dextran 20% solution	**Riboflavin solution**	Dextran-free riboflavin 0.1% solution
Every 2 mins for 30mins	**Duration of soak**	Every 2mins for 10mins
UV-X, Peschke Meditrade GmbH, Huenenberg, Switzerland	**UV illumination device**	KXL, Avedro, Inc., Waltham, Massachusetts, MA, USA
3mW/cm^2^ for 30 minutes (total energy: 5.4J/cm^2^).	**Illumination protocol**	30 mW/cm^2^ continuously for 4 minutes (total energy: 7.2J/cm^2^).

**Table 2:** Comparison of baseline characteristics in CXL and KXL group.

	**CXL (N=29)**	**KXL (N=47)**	**P value**
Age (years)	29 +/- 7	28 +/- 7	0.452
Sex (Male: Female)	21:8	37:10	0.530
Follow up (months)	13.10 +/- 3.30	12.20 +/- 2.70	0.191
UCVA	0.86 +/- 0.40	0.80 +/- 0.30	0.400
BCVA	0.37 +/- 0.30	0.40 +/- 0.20	0.703
Spherical equivalent (D)	-4.72 +/- 3.60	-4.30 +/- 3.00	0.591
Cylinder (D)	-4.94 +/- 3.40	-5.50 +/- 2.10	0.434
K1 (D)	47.68 +/- 4.30	47.40 +/- 5.00	0.808
K2 (D)	52.29 +/- 5.40	52.15 +/- 5.30	0.915
Kmean (D)	49.82 +/- 4.50	49.78 +/- 5.00	0.969
Central corneal thickness (µm)	491.52 +/- 46.30	492.64 +/- 31.20	0.909
Minimal corneal thickness (µm)	460.10 +/- 44.90	467.78 +/- 31.00	0.425
Corneal hysteresis (mm Hg)	7.82 +/- 1.50	8.23 +/- 1.60	0.273
Corneal resistance factor (mm Hg)	6.38 +/- 1.40	6.28 +/- 1.70	0.790
Endothelial cell density (Cells/mm^2^)	2860 +/- 368	3146 +/- 544	0.017

Abbreviations and units: UCVA, uncorrected visual acuity; BCVA, best corrected visual acuity; D, Diopter

**Table 3:** Refractive changes before and after CXL and KXL.

	**Baseline**	**1 Month**	**3 Months**	**6 Months**	**12 Months**
**Spherical Equivalent (D)**					
**CXL**	-4.72 +/- 3.6	-3.71 +/- 3.6 (P=0.091)	-4.06 +/- 4.5 (P=0.434)	-3.93 +/- 4.03 (P=0.165)	-3.82 +/- 4.4 (P=0.247)
**KXL**	-4.30 +/- 3.1	-5.25 +/- 4.0 (P=0.046)	-5.08 +/- 4.6 (P=0.019)	-4.79 +/- 3.7 (P=0.379)	-5.11 +/- 4.07 (P=0.131)
**Cylinder (D)**					
**CXL**	-4.94 +/- 3.4	-3.93 +/- 2.7 (P=0.048)	-4.14 +/- 3.1 (P=0.129)	-4.27 +/- 2.8 (P=0.544)	-4.21 +/- 3.5 (P=0.490)
**KXL**	-5.5 +/- 2.1	-5.7 +/- 2.1 (P=0.013)	-5.77 +/- 2.1 (P=0.009)	-5.73 +/- 2.1 (P=0.026)	-5.82 +/- 2.15 (P=0.002)

Abbreviations and units: D, Diopter

**Table 4:** Review of studies comparing conventional and accelerated crosslinking.

**Study**	**Study design**	**Conventional crosslinking**		**Accelerated crosslinking**		**Follow up** **(months)**	**Findings**
		**N**	**Protocol/ platform**	**N**	**Protocol/ platform**		
Kanellopoulos, 2012	Prospective randomized comparative case series	21	3mW/cm^2^ for 30 mins	21	7mW/cm^2^ for 15 mins	46	Comparable changes in visual acuity, refraction, reduction in steepest K, no progression in both groups
Cinar **et al*.* 2014	Prospective comparative case series	13	3mW/cm^2^ for 30 mins	13	9mW/cm^2^ for 10 mins	6	Comparable visual and refractive results, decrease in Km and Kmax in both groups
Brittingham **et al*.* 2014	Retrospective case series	81	3mW/cm^2^ for 30 mins UV-X 1000	50	9mW/cm^2^ for 10 mins UV-X 2000	12	Increased steepening of anterior cornea in accelerated (+0.72D) compared to conventional group (-0.76D)
Ng **et al*.* 2015	Retrospective comparative case series	14	3mW/cm^2^ for 30 mins UV-X 1000	12	9mW/cm^2^ for 10 mins UV-X 2000	12	Greater reduction in Kmax and Kmean in conventional group compared to accelerated group
Hashemi **et al*.* 2015	Prospective randomized comparative case series	31	3mW/cm^2^ for 30 mins UV-X	31	18mW/cm^2^ for 5 mins UV-X	6	Comparable visual acuity, refractive, keratometric and biomechanical outcomes
Chow **et al*.* 2015	Prospective comparative case series	19	3mW/cm^2^ for 30 mins UV-X	19	18mW/cm^2^ for 5 mins CCL-VARIO	12	Comparable visual acuity and refractive outcomes. More topographic flattening in the conventional group compared to accelerated group
Hashemian **et al*.* 2014	Prospective randomized controlled trial	153	3mW/cm^2^ for 30 mins CCL-VARIO	77	30mW/cm^2^ for 3 mins CCL-VARIO	15	Comparable changes in visual acuity, refraction, endothelial cell density, Kmax, anterior stromal keratocyte density and subbasal nerve density
Sherif, 2014	Prospective randomized comparative study	11	3mW/cm^2^ for 30 mins UV-X	14	30mW/cm^2^ for 4 mins and 20s Avedro KXL	12	Comparable reduction in Kmax, changes in corneal hysteresis, corneal resistance factor and central corneal thickness
Tomita *et al.* 2014	Prospective comparative study	18	3mW/cm^2^ for 30 mins CCL VARIO	30	30mW/cm^2^ for 3 mins Avedro KXL	12	Comparable changes in visual acuity, refractive, keratometric readings, biomechanical responses between the 2 groups
Shetty **et al*.* 2015	Prospective randomized interventional study	36	3mW/cm^2^ for 30 mins Avedro KXL	36	9mW/cm^2^ for 10 mins	12	Conventional group and accelerated groups with irradiance of 9mW/cm2 and 18mW/cm^2^ showed better visual, refractive and tomographic improvements. Minimal stabilization of disease in 30mW/cm^2^ group
				33	18mW/cm^2^ for 5 mins		
				33	30mW/cm^2^ for 3 mins Avedro KXL for all groups		
